# Reply on comment on “Thinner temporal peripapillary retinal nerve fibre layer in Stargardt disease detected by optical coherence tomography”

**DOI:** 10.1007/s00417-021-05301-1

**Published:** 2021-08-17

**Authors:** Michael Reich, Wolf A. Lagrèze

**Affiliations:** Eye Center, Medical Center - University of Freiburg, Faculty of Medicine, University of Freiburg, Killianstrasse 5, D-79106 Freiburg im Breisgau, Germany

We thank Dr. Pradeep Kumar Panigrahi for his comment on our article “Thinner temporal peripapillary retinal nerve fibre layer in Stargardt disease detected by optical coherence tomography” [1] and for his valuable suggestions. We agree that the study design is better described by “cross-sectional, monocentric, observational study”. Regarding the influence of possible systemic medication, we can confirm that none of the included patients was taking systemic medications associated with peripapillary retinal nerve fibre layer thinning.
